# Application of Composite Dictionary Multi-Atom Matching in Gear Fault Diagnosis

**DOI:** 10.3390/s110605981

**Published:** 2011-06-03

**Authors:** Lingli Cui, Chenhui Kang, Huaqing Wang, Peng Chen

**Affiliations:** 1 Key Laboratory of Advanced Manufacturing Technology, Beijing University of Technology, Chao Yang District, Beijing, 100124, China; E-Mails: acuilingli@163.com (L.C.); kavie0331@sina.com (C.K.); 2 School of Mechanical & Electrical Engineering, Beijing University of Chemical Technology, Chao Yang District, Beijing, 100029, China; 3 Graduate School of Bioresources, Mie University, 1577 Kurimamachiya-cho, Tsu, Mie, 514-8507, Japan; E-Mail: chen@bio.mie-u.ac.jp

**Keywords:** composite dictionary, multi-atom matching, threshold de-noising, segmented decomposition and reconstruction, gear fault diagnosis, genetic algorithm

## Abstract

The sparse decomposition based on matching pursuit is an adaptive sparse expression method for signals. This paper proposes an idea concerning a composite dictionary multi-atom matching decomposition and reconstruction algorithm, and the introduction of threshold de-noising in the reconstruction algorithm. Based on the structural characteristics of gear fault signals, a composite dictionary combining the impulse time-frequency dictionary and the Fourier dictionary was constituted, and a genetic algorithm was applied to search for the best matching atom. The analysis results of gear fault simulation signals indicated the effectiveness of the hard threshold, and the impulse or harmonic characteristic components could be separately extracted. Meanwhile, the robustness of the composite dictionary multi-atom matching algorithm at different noise levels was investigated. Aiming at the effects of data lengths on the calculation efficiency of the algorithm, an improved segmented decomposition and reconstruction algorithm was proposed, and the calculation efficiency of the decomposition algorithm was significantly enhanced. In addition it is shown that the multi-atom matching algorithm was superior to the single-atom matching algorithm in both calculation efficiency and algorithm robustness. Finally, the above algorithm was applied to gear fault engineering signals, and achieved good results.

## 1. Introduction

Gears are important components in rotating machinery, and fault detection and diagnosis of gears has been the subject of intensive investigation. Generally, gear fault vibration signals heavily corrupted by noise are non-stationary signals whose fault features are more difficult to successfully extract than stationary signals. Therefore, studies on such signals are of extreme significance to engineering applications.

Fault diagnosis of gears is currently a topic of intensive study, and many time-frequency analysis methods based on vibration signal have been developed, which include Short-time Fourier transform, Wigner-Ville distribution, Wavelet transform and Hilbert-Huang transform, *etc.*, and good results have been achieved using these in gear fault diagnosis [1–7]. However, general time-frequency analysis methods lack adaptability to complicated signals due to the singleness of their basic decomposition functions. To achieve a more flexible, concise and adaptive expression of signals, Mallat and Zhang summed up previous research achievements based on wavelet analysis and raised the idea of decomposing signals on the over-complete dictionary in 1993. The basic function was replaced by the over-complete redundant function that was known as the atom dictionary, and the elements in the atom dictionary were known as atoms. M atoms with the optimal linear combination were picked out from the atom dictionary to represent a signal, which was known as the Sparse Approximation of the signal [8]. Besides, a matching pursuit (MP) algorithm based on the time-frequency atom dictionary was proposed. The algorithm adopted a strategy of obtaining the sparse expression of signals through gradual approximation. A group of primitive functions, *i.e.*, atoms, were selected from the atom dictionary to calculate a linear expansion of signals and to achieve the successive approximation of signals by solving the rectangular projection of the signals on each atom.

The proposal of the sparse decomposition based on the MP algorithm aroused extensive interest among researchers who did a lot of work on the optimization and improvement of the algorithm, as well as the expansion of its fields of application. In terms of the atom dictionary construction, atom dictionary construction methods based on the Gabor function [9,10], wavelet and wavelet packet functions [11–13], impulse time-frequency atoms [14], multi-scale chirplet [15] and other function models were proposed. In terms of applications, the MP algorithm has wide applications in mechanical failure diagnosis [15–19], image processing, video decoding [10], *etc.* Aharon *et al.* proposed a K-SVD optimization algorithm in which a redundant atom dictionary with superior sparse expression effects was obtained through an iterative learning and optimization process performed on the initial atom dictionary [20]. Wang *et al.* introduced characteristic signal waveforms as the basic atoms for the atom dictionary construction and applied them to the identification of the fault modes of rolling bearings [21]. Feng *et al.* applied various atom decomposition methods, including framework decomposition, the best orthogonal basis, MP, and basis pursuit, in the extraction of gear fault characteristics, and compared the effects of applying different methods [19]. Some other scholars have studied the influences of different iteration termination conditions in the MP algorithm on the decomposition effects of high-noise signals [22].

However, all the references above have focused on the MP algorithms and applications using single-atom dictionaries, and few papers on composite dictionaries with different atom dictionaries being combined were reported. The concept of composite dictionaries was proposed in [16]. However, no further investigation and studies have been conducted, to the best of our knowledge. In this paper a new sparse decomposition and reconstruction algorithm is proposed, based on the Composite Dictionary Multi-atom matching pursuit in which the threshold de-noising was introduced. The new algorithm has a favorable effect in the extraction of the impulse signals of gear faults. Aiming at examining the effects of data lengths on the calculation efficiency of the algorithm, an improved algorithm of segmented decomposition and reconstruction that reduced calculation and program running time was proposed and the effect of noise on the algorithm stability was discussed. The analysis results of gear fault simulation signals and engineering signals indicate the feasibility and validity of the method.

The paper is organized as follows: Section 2 presents the concept of sparse decomposition based on the MP algorithm. Sections 3 and 4 address the specific decomposition and reconstruction algorithm of composite dictionary multi-atom matching and its operational details for gear faults. Section 5 presents simulation signal analysis results and a segmented decomposition algorithm for long signal process is given in Section 6. The algorithm is validated through an application example in Section 7. Finally, Section 8 concludes the paper with some remarks about possible future work.

## 2. Sparse Decomposition Based on the MP Algorithm

For a given set *D* = {***g****_k_*, *K =* 1, 2,...,*K*}, the elements are unit vectors in the whole Hilbert space, *i.e.*, *H* = *R^N^*, where *K ≫ N*. The set *D* is known as an atom dictionary, and the elements are known as atoms. Because of the redundancy of an atom dictionary (*K ≫ N*), vector ***g****_k_* is no longer linearly independent. For an arbitrary real signal *f* ∈*H* with the length of *N*, a linearcombination composed of *m* atoms is found in *D* :

(1) $$f\;=\;\sum_{m=0}^{M-1}c_{k_{m}}g_{k_{m}}$$

Equation (1) is the sparse decomposition of signals, or known as the sparse expression. MP is an algorithm that is frequently used for sparse decomposition. The principle is as follows:

Supposing that *H* represents the Hilbert space, then the atom dictionary in *H* is defined, **‖*g****_k_***‖** = 1. Let *f* ∈*H.* To approach *f* through MP, the most suitable one is first selected from the over-complete atom dictionary, *i.e.*, Equation (2) is satisfied:

(2) $$\left|<f,\;g_{k_{0}}>|\;=\;\text{sup}|<f,\;g_{k}>\right|$$

Thus, signal *f* can be decomposed into the following form:

(3) $$f\;=\;<f,\;g_{k_{0}}\;>\;g_{k_{0}}\;+\;R^{1}f$$

It is clear that *g*_*k*_0__ and *R*^1^ *f* are orthogonal. Let 〈*g_k_*, *f*〉 = < *f*, *g_k_* > *g_k_*, then Equation (4) can be obtained:

(4) $${\left||f|\right|}^{2}\;=\;|〈g_{k_{0}},\;f〉{|}^{2}\;+\;{\left||R^{1}f|\right|}^{2}$$

To minimize the energy ‖*Rf*‖ for approaching errors, *g*_*k*_0__ ∈ *H* is necessary to maximize **|**< *f*, *g*_*k*_0__ >**|**. Under infinite dimensions, it is generally impossible to acquire the extreme value of |< *f*, *g_k_* >|, and it is only feasible to select an approximately optimal atom *g*_*k*_0__, therefore:

(5) $$\left|<f,\;g_{k_{0}}>|\geq \;\alpha \;\text{sup}|<f,\;g_{k}>\right|$$

where *α* is the optimization factor that satisfies 0 < *α* ≤1. Under finite dimensions, the maximum of |< *f*, *g_k_* >| exists. Here *α* = 1

Then, the same step is performed on the residual *R*^1^ *f*, and Equation (6) is obtained:

(6) $$R^{1}f=〈g_{k_{1}},R^{1}f〉+R^{2}f$$

Satisfying:

(7) $$\left|<R^{1}f,g_{k_{1}}>|=\text{sup}|<f,g_{k}>\right|$$

The MP algorithm is an iterative process. Signal residual *Rf* continues to be decomposed by constantly projecting it to a vector in the atom dictionary that matches it best. Thus, after *m*+1 times of iteration:

(8) $$R^{m}f=〈R^{m}f,g_{k_{m}}〉+R^{m+1}f$$

where *g*_*k_m_*_ satisfies:

(9) $$|〈R^{m}f,g_{k_{m}}〉|=\text{sup}|〈R^{m}f,g_{k}〉|$$

Because *R^m+^*^1^ *f* and *g*_*k_m_*_ are orthogonal, then:

(10) $${\left\|R^{m}f\right\|}^{2}={\left|<R^{m}f,g_{k_{m}}>\right|}^{2}+{\left\|R^{m+1}f\right\|}^{2}$$

With the decomposition process above being executed to order *n* :

(11) $$f=\sum_{m=0}^{M-1}<R^{m}f,g_{k_{m}}>g_{k_{m}}+R^{M}f$$

Similarly, energy ‖*f*‖^2^ can also be decomposed into the following summation form:

(12) $${\left\|f\right\|}^{2}=\sum_{m=0}^{M-1}{\left|<R^{m}f,g_{k_{m}}>\right|}^{2}+{\left\|R^{M}f\right\|}^{2}$$

According to Equation (12), the *M*-order approximation form of vector ***f*** in atom dictionary *D* can be obtained. The approximation error is expressed as *R^M^* *f*. It is clear that the error energy trends to gradually attenuate with the decomposition.

## 3. Decomposition and Reconstruction Algorithm of Composite Dictionary Multi-Atoms Matching

A key step to implement the MP algorithm is how to construct an atom dictionary, so that signals can better match atoms during the sparse decomposition. The construction of the atom dictionary directly affects the sparse expression of the signals to be analyzed. The atom dictionary construction method based on the parameterized function model is the most frequently adopted. A specific primitive function is discretely parameterized (e.g., time, frequency, contraction, translation, modulation, *etc.*) in this method. Each parameter group corresponds to an atom, and the set of atoms constitutes the atom dictionary. A dictionary based on a specific function structure is only suitable for analyzing signals with certain characteristics; however, the signals in engineering have complicated components. To effectively extract the abundant characteristic information contained in signals, different characteristic atom dictionaries are combined to form a composite dictionary according to the structural characteristics of signals [9]. During the sparse decomposition of signals, a best matching atom is selected from each characteristic dictionary each iteration, with their projections being solved respectively. The weighted linear superposition of the projections is used as the overall projection. The best matching atom refers to the one with the maximal absolute coefficient of the projection that the signal projects on it. The projection coefficient can be obtained by calculating the inner product of the signal and the atom, *i.e.*, *c* = < *x*,*d* >, where *x* represents the signal; *d* represents the atom, and **‖***d***‖** = 1. The signal projection on the atom can be expressed as the product of the projection coefficient and the atom.

The introduction of the above idea is known as the composite dictionary multi-atoms matching. The decomposition algorithm is implemented as follows:

1. Corresponding characteristic functions are selected to construct atom dictionary ***D****_i_* (*i* = 1,2,...,*I; I* is the number of characteristic atom dictionaries) in a parameterized manner according to the structural characteristics of the signals to be analyzed. Two or more characteristic atom dictionaries are combined to form composite dictionary ***D***.
2. Primary signal *x*(*t*) is given to the residual signal to obtain initial residual *r*_0_.
3. For residual signal *r_m_* (*m* = 0,1,2,...,*M*-1; *M* is the times of iteration), a best matching atom *d_mi_* is searched in corresponding characteristic dictionary ***D****_i_* respectively, and the projection coefficient *c_mi_* is solved. The weighted linear superposition of all the projections are the overall projection, *i.e.*,
  (13) $$p_{m}=\sum_{i=1}^{I}a_{i}c_{mi}d_{mi}$$
  where *a_i_* is the weighted coefficient of each characteristic atom dictionary, and $\sum_{i=1}^{I}a_{i}=1$.
4. With the residual signal being subtracted by the overall projection, a new residual signal is obtained.
5. Steps (3–4) are iterated till the iteration termination condition is satisfied.
6. After the sparse decomposition, matching coefficient *c_mi_* and matching atom *d_mi_* of each order are obtained.

**Figure 1.**
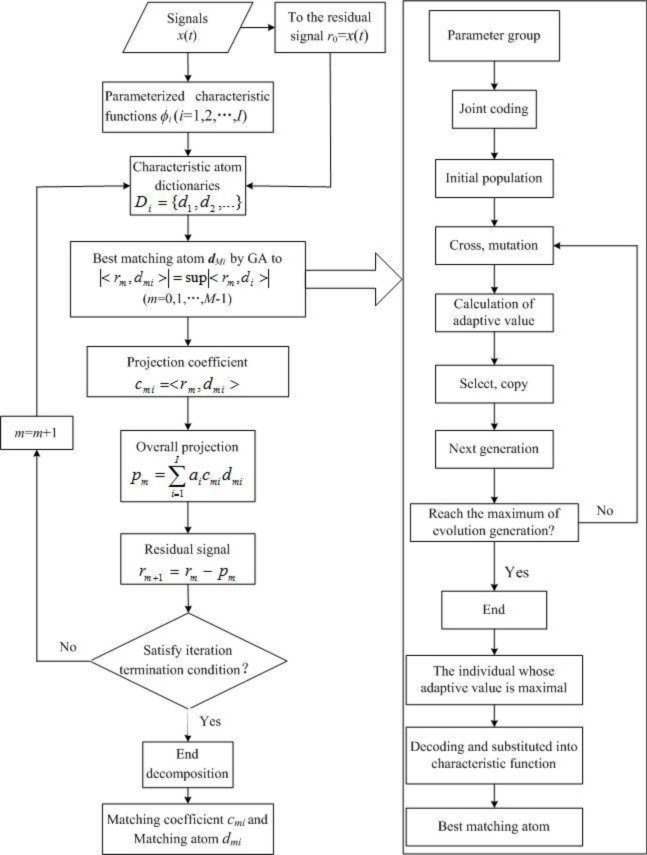
Flow chart of the decomposition algorithm.

The corresponding flow chart is shown in Figure 1.

The reconstruction algorithm is an inverse process of the decomposition algorithm. Equation (14) is as follows:

(14) $$\tilde{x}=\sum_{m=1}^{M}\sum_{i=1}^{I}a_{i}c_{mi}d_{mi}$$

The principle of threshold de-noising is introduced in Equation (14). For each reconstruction calculation instance, the projection coefficient *c_mi_* is set to be zero if it is less than some group of thresholds *T_i_* For those being more than or equal to the thresholds, they remain unchanged (hard threshold) or get weakened (soft threshold), *i.e.*,

(15) $${\tilde{c}}_{mi}=h_{t}(c_{mi})=\{\begin{array}{l} c_{mi},|c_{mi}|\geq T_{i}\\ 0,|c_{mi}|<T_{i} \end{array}$$

or:

(16) $${\tilde{c}}_{mi}=s_{t}(c_{mi})=\{\begin{array}{l} c_{mi}-T_{i},c_{mi}\geq T_{i}\\ c_{mi}+T_{i},c_{mi}\leq -T_{i}\\ 0,|c_{mi}|<T_{i} \end{array}$$

The calculation equation of the threshold is $T_{i}=\sigma_{i}\sqrt{2\text{ln}M_{i}}$, where σ*_i_* is the standard difference of matching coefficients of all orders; and *M_i_* is the number of matching coefficients, *i.e.*, the times of iteration. Then, the calculation equation for the reconstruction algorithm after the threshold is:

(17) $$\tilde{x}=\sum_{m=1}^{M}\sum_{i=1}^{I}a_{i}{\tilde{c}}_{mi}d_{mi}$$

## 4. Composite Dictionary Multi-Atoms Matching for Gear Fault

The vibration signals of such typical rotating machinery such as gears are basically induced by the meshing effect and the rotation of gears, and the characteristics of shock vibration and transient vibration may also emerge in the vibration signals of fault gears. To achieve the effective matching analysis on the characteristic structures of gear vibration signals, the Fourier dictionary and the impulse time-frequency dictionary [14] are constructed using the method of parameterized function model, and the two dictionaries are combined to form a composite dictionary. The construction method is described in detail as follows: the primitive function of the Fourier dictionary is a sine function, *i.e.*,

(18) $$\phi_{fou}(f,\gamma )=K_{fou}\text{sin}(2\pi ft+\gamma )$$

where *f* is the frequency parameter; *γ* is the phase parameter; and *K* *_fou_* is the normalized coefficient. To guarantee that each atom has the unit energy, *i.e.*, **‖***ϕ_fou_* (*f*, *γ*)**‖**_2_ = 1, the primitive function of the impulse time-frequency dictionary refers to the exponential decay function, *i.e.*,

(19) $$\phi_{imp}(p,u,f,\Phi )=\{\begin{array}{l} K_{imp}e^{-p(t-u)}\text{sin}2\pi f(t-\Phi ),t\geq u\\ 0,t<u \end{array}$$

where *p* is the damping characteristic of the impulse response; *u* is the initial time when an impulse response event occurs; *f* is the damped natural frequency of the system; *Φ* is the phase deviation; and *K_imp_* is the normalized coefficient. Discrete values are given to parameters in the function model within a certain range. A group of parameters are substituted into the function model to obtain an atom, and all of the atoms form a dictionary. However, there are too many parameters in models (two in the Fourier dictionary and four in the impulse time-frequency dictionary) and it is very difficult to evaluate them one by one. Therefore, the optimization algorithm is used to find the optimal marching atom.

The genetic algorithm (GA), a kind of calculation model of biological evolution simulating of natural selection and genetic mechanism based on Darwin’s theory of evolution, was proposed by Holland in 1975. It is a method of searching the optimal solution by simulating the natural evolutionary process [23]. In this algorithm, joint coding is first performed on all the parameter groups needed for constructing characteristic atom dictionaries, to produce randomly an initial population with a certain scale *N*. Each parameter group corresponds to an individual, and crossing and mutation are conducted according to a certain probability. The fitness value of each individual is then calculated, those best individuals with the maximum fitness directly go to the next generation, *N*-1 individuals are selected from the parent generation with the random iteration method and go to the next generation, and all the next-generation individuals form the new population. The new population repeats crossing, mutation, fitness calculation, selection and other operations to continuously evolve, till the evolution generations reach a preset value. Finally, an individual with the maximum fitness is selected from the optimal ones in each generation as the optimal parameter group, and is substituted into the primitive function after decoding to form the optimal matching atom. The corresponding flow chart is shown in Figure 1.

## 5. Simulation Signal Analysis

In Reference [24], a vibration signal model for gears with cracking faults was presented:

(20) $$y(t)=\sum_{m=0}^{M}A_{m}[1+{\tilde{a}}_{m}(t)]\text{cos}\{2\pi f_{m}t+\beta_{m}+{\tilde{b}}_{m}(t)\}+d(t)\text{cos}(2\pi f_{r}t+\theta_{r})$$

**Figure 2.**
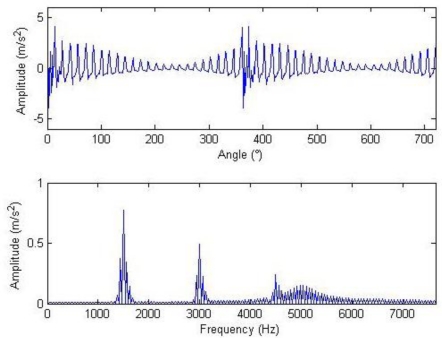
Signal waveform and frequency spectrum.

**Figure 3.**
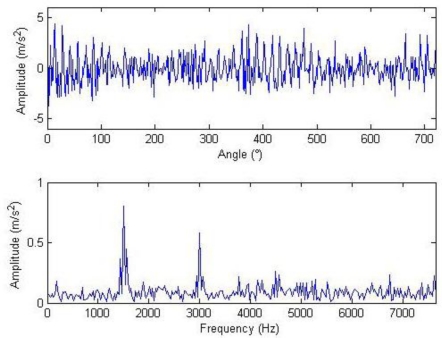
Signal waveform and frequency spectrum after noising.

The meanings of the various parameters in Equation (20) can be known by referring to the original literatures. During the simulation, the gear is assumed to have 25 teeth and the rotation frequency is 60 Hz. The meshing vibration has three rather significant meshing harmonics. The meshing frequencies of the three harmonics are 1.5, 3 and 4.5 kHz. To simulate the gear fault effects, an impact is assumed to be produced by the gear tooth cracking near 0° in a complete revolution of gear and the impact excites the resonance at 5 kHz. The sampling frequency is set to be 15,360 Hz, the time-domain waveform and spectra upon two rounds of gear rotation are then shown in Figure 2. From the frequency spectrogram, meshing frequencies of the three orders (1.5, 3 and 4.5 kHz), their modulation sidebands and the impulse response bands in the vicinity of 5 kHz can be clearly found. To better fit real fault signals, random noise in standard normal distribution is introduced. The signal to noise ratio (*SNR*) is −0.72 dB as shown in Equation (21). The waveform and the frequency spectra are shown in Figure 3. The system resonance band induced by the fault shock after noising is basically overwhelmed by the noise:

(21) $$SNR=20{\text{log}}_{10}(v_{s}/v_{n})$$

where *v_s_* and *v_n_* are the effective values of the primary simulation signal and the noise, respectively.

**Figure 4.**
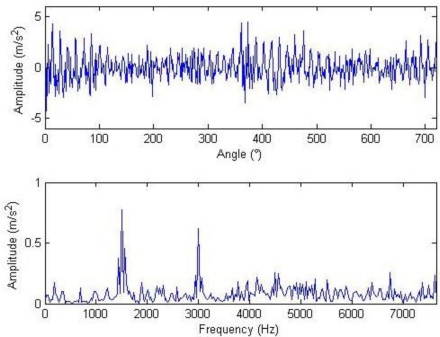
Waveform and frequency spectrum of reconstructed signals.

Composite dictionary multi-atom matching decomposition and reconstruction were performed on the simulation signal corrupted by noise using the algorithm. Experiments show that parameter setting in GA has a great effect on optimization results and computational efficiency. After balancing these two factors, the analysis parameters are as follows: in the Fourier dictionary, the range of *f* is set to be 1–8,192 Hz; range of *γ* is 0–2π; joint coding length is 20; the population is 200; the maximal number of evolution generations is 100; the single-point mode is adopted for crossing, with 0.6 as the crossing probability; single-point mutation is adopted with the probability of 0.1; and the length of atom is 512. In the impulse time-frequency dictionary, the range of *p* is set to be 1,001–2,024; range of *f* is 4,001–6,048 Hz; range of *u* is 1–512; setting *Φ* = 0 to simplify the problem, joint coding length is 30; the population is 300; and the other parameters are consistent with the Fourier dictionary. The terminal condition for MP iteration is the ratio of the residual signal energy to the initial one, *i.e.*, *η* < 0.1. The weighted coefficients of the impulse time-frequency dictionary and the Fourier dictionary, *i.e.*, both *a*_1_ and *a*_2_, are equal to 0.5. The waveform and the frequency spectrum after the reconstruction are shown in Figure 4. Compared with Figure 3, the algorithm has clearly favorable reconstruction precision.

**Figure 5.**
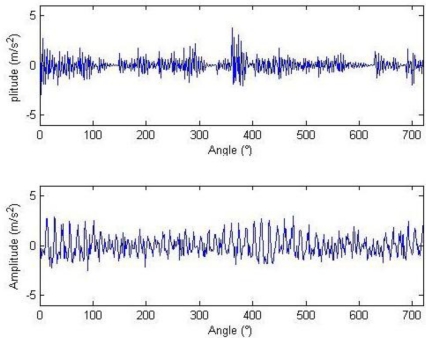
Impulse component (above) and harmonic component (below).

In the reconstruction algorithm, the weighted coefficients of the impulse time-frequency dictionary and the Fourier dictionary changed. For instance, it is set that *a*_1_ = 1, *a*_2_ = 0 or *a*_1_ = 0, *a*_2_ = 1. Here, the signals reconstructed only through impulse time-frequency atoms are known as impulse components while those reconstructed only through Fourier atoms are known as harmonic components. Thus, the impulse components or the harmonic components can be separately extracted, as shown in Figure 5. Figure 4 clearly shows that there are two large but not significant impulses, and the impulse interval cannot be determined.

**Figure 6.**
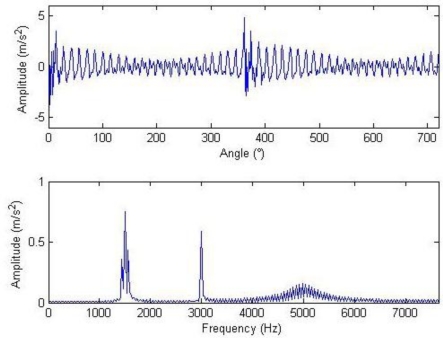
Waveform and frequency spectrum harmonic of reconstructed signals with hard threshold.

**Figure 7.**
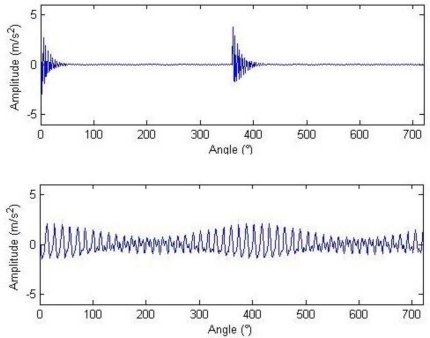
Impulse component (above) and component (below) with hard threshold.

From the equation for calculating the threshold, it can be seen that the soft threshold only has the effect of amplitude attenuation different from the hard threshold. Therefore, the hard threshold is adopted here. The waveform and frequency spectrum of the reconstructed signals after the threshold are shown in Figure 6. Most noise has been removed, and the waveforms after two cycles of gear rotation as well as the impulse at 0° can be seen. The meshing frequency at 1.5 and 3 kHz as well as the system resonance band induced by the modulation sideband and the impulse can also be seen from the frequency spectrogram. Similarly, setting that *a*_1_ = 1, *a*_2_ = 0 or *a*_1_ = 0, *a*_2_ = 1, then the impulse component and the harmonic component can be extracted respectively, as shown in Figure 7.

**Figure 8.**
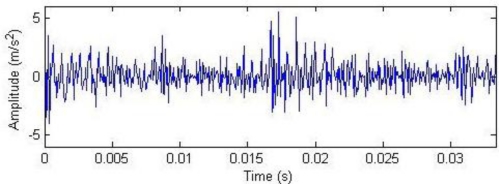
Reconstructed signals with single-atom atom matching without threshold.

**Figure 9.**
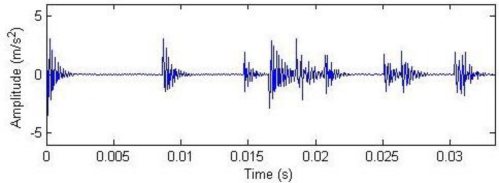
Reconstructed signals with single- matching and hard threshold.

For signals corrupted by noise in Figure 3, reconstructed signals without threshold or with hard threshold are shown in Figures 8 and 9, provided that only the impulse time-frequency dictionary is used for single-atom matching. They are compared with Figure 5(above) and Figure 7(above). Compared with those reconstructed with impulse time-frequency dictionary single-atom matching, impulse signals can be extracted more effectively with the impulse component reconstructed with the composite dictionary multi-atoms matching, and the effect of threshold de-noising is better. In the running of MATLAB, the total iteration of composite dictionary multi-atoms matching is 50, and the running period of the decomposition algorithm is 128 s. The total iteration of impulse time-frequency dictionary single-atom matching is 393, and the running of the decomposition algorithm lasts 686 s. Compared with single dictionary, a composite dictionary can better match the signals to be analyzed in structure, so that the decomposition efficiency can be significantly enhanced.

To illustrate the influences of multi-atom matching, single-atom matching and threshold or non-threshold on the extraction effects of impulse signals, kurtosis indices and impulse indices are introduced. Their definitions are in Equations (21) and (22), both of which can reflect the amplitudes of the impulse energy in signals. The more the characteristic value is, the more distinct the fault information is, and the more significant the extracted impulse signals become. The primary signals with noise, the impulse component treated with multi-atom matching without threshold (multi-atom without threshold), the impulse component treated with multi-atom matching hard threshold (multi-atom hard threshold), reconstructed signals treated with single-atom matching without threshold (single-atom without threshold) and reconstructed signals treated with single-atom matching hard threshold (single-atom hard threshold) are calculated, respectively. The signals are described in Figures 3, 5 and 7, Figures 8 and 9. The calculation results are shown in Table 1. It is clear that better effects of impulse signal extraction are achieved with the multi-atoms matching and the hard threshold compared with the single-atom matching and without threshold, respectively.

The kurtosis index is defined as:

(22) $$K=\frac{\frac{1}{N}\sum_{i=1}^{N}x_{i}^{4}}{\phi_{x}^{4}}$$

The impulse index is defined as:

(23) $$I=\frac{\text{max}|x(i)|}{\mu_{\left|x\right|}}$$

where *ϕ_x_* is the root mean square of a signal; and *μ***_|_**_x_**_|_** is the mean absolute value of a signal.

Intensified noise is applied to the simulation signals above. When the *SNR* of noising signals is −0.72, −6.60 and −10.21dB, kurtosis indices and impulse indices are used to discuss the effects of the composite dictionary multi-atom matching and the impulse time-frequency dictionary single-atom matching on the impulse signal extraction. The comparison results are listed in Table 2. The comparison between the kurtosis and impulse indices with noising signals shows that the extraction effects of the multi-atom matching and the corresponding hard threshold are still significantly enhanced while there is little enhancement in the effects of the single-atom matching and the corresponding hard threshold.

## 6. Segmented Decomposition Algorithm for Long Signal Process

The frequency spectrogram in Figure 6 shows the system resonance band induced by impulse faults. However, the frequency resolution is not high enough, showing very few signal points (the length *N* of the signal to be analyzed in Section 4 is 512). The minimal interval on the frequency axis is fs/*N* = 30 Hz, which causes inexact modulation frequency identification for the sideband. To enhance the frequency resolution, the data are increased. However, this causes the iteration of the decomposition algorithm, increases the time needed for each iteration, and results in overlong running of the MATLAB program. Table 3 shows the iteration times and the calculation time of the decomposition algorithm when the number of data points is 512, 1,024, and 2,048.

To solve the problem of the running time of the program, the algorithm is improved. The primary data sequence is evenly segmented by 512, and each section is decomposed to obtain the matching coefficients and matching atoms of all orders for each section of the data sequence. The threshold is also conducted upon sections, respectively. After the reconstruction, all sections are combined to form the reconstructed signal. Thus, the running time of the improved algorithm is significantly reduced. Table 3 shows the comparison results. The iteration of the initial decomposition algorithm is doubled if the number of data points is doubled, while the running time is quadrupled. However, both the total iteration and the running time of the segmented decomposition algorithm are only doubled.

**Figure 10.**
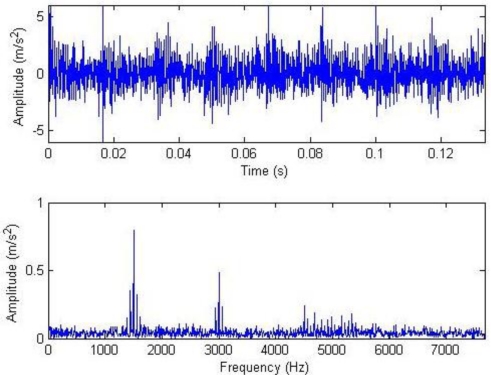
Waveform and frequency spectrum of primary signals (2,048).

**Figure 11.**
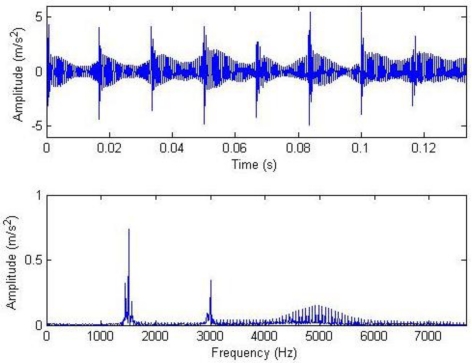
Waveform and frequency spectra of reconstructed signals after hard threshold (2,048).

**Figure 12.**
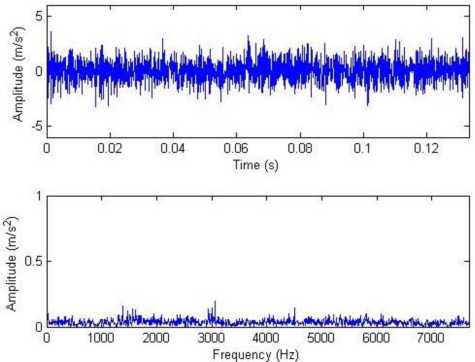
Waveform and frequency spectrum of signals after filtration with hard threshold.

Figures 10–12 show the waveform and frequency spectra of primary signals, the waveform and frequency spectra of the reconstructed signals with hard threshold, and the waveform and frequency spectra of the signals removed through filtration by threshold when the number of data points is 2,048, respectively. It can be seen that most noise signals are removed by filtration through hard threshold.

**Figure 13.**
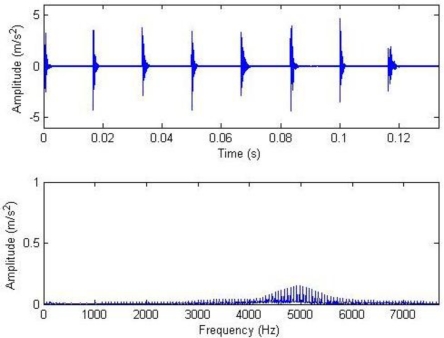
Impulse component with hard threshold (2,048).

**Figure 14.**
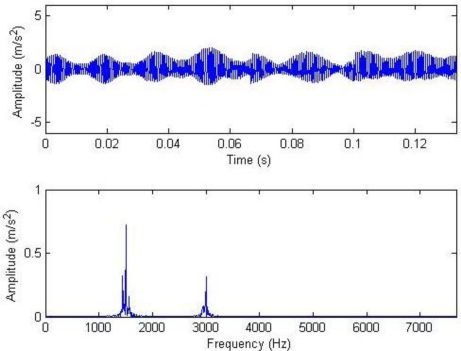
Harmonic component with hard threshold.

**Figure 15.**
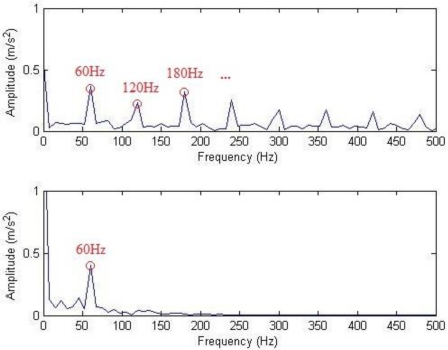
Demodulation spectra.

The waveforms and frequency spectra of the separately extracted impulse component and harmonic component are shown in Figures 13 and 14, respectively. The impulse component is embodied in eight times of significant equally-spaced shock, with an interval of 0.0167 s, and the frequency is exactly the rotation frequency, *i.e.*, 60 Hz. The frequency spectra are embodied in the system resonance frequency band induced by the fault shock (in a vicinity of 5 kHz). The harmonic frequency spectra are embodied by the meshing frequencies and modulation sidebands of all orders (some sidebands are removed by filtration due to low amplitudes). The demodulation spectra of the impulse component and the harmonic component are shown in Figure 15. Figure 15 (above) shows that the demodulation spectrum of the impulse component with a rotation frequency of 60 Hz and harmonic frequencies of all orders can be clearly found. Figure 15 (below) shows that the demodulation spectrum of the harmonic component and the harmonic frequencies of high orders are removed by filtration during the hard threshold with only the rotation frequency of 60 Hz being displayed.

## 7. Application Example

**Figure 16.**
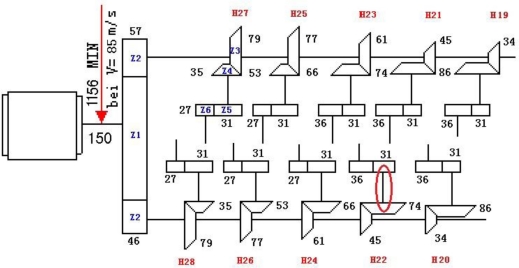
Driving chain of gearbox.

Figure 16 shows the driving chain of a gearbox on a high-speed finishing mill from a steel plant. On-spot monitoring information indicated that modulation information had been reflected by the frequency spectrogram about the vibration data since 14 September 2006. The modulation frequency was the rotation frequency of shaft II on the bevel box of finishing support H22 (30.1 Hz), as marked in Figure 16. This phenomenon remained at later periods.

**Figure 17.**
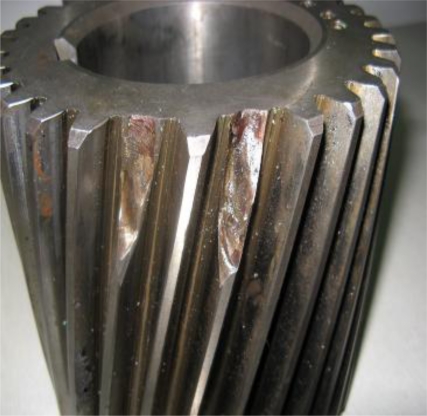
Fault gear Z5 on shaft II of the bevel box.

**Figure 18.**
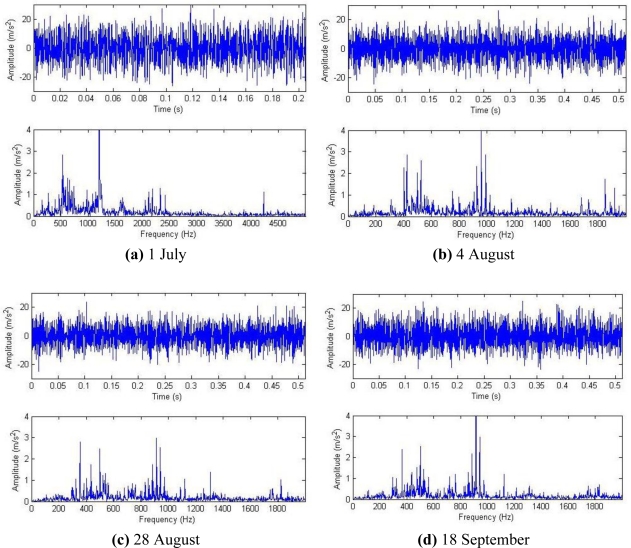
Waveforms and frequency spectra of historical data.

**Figure 19.**
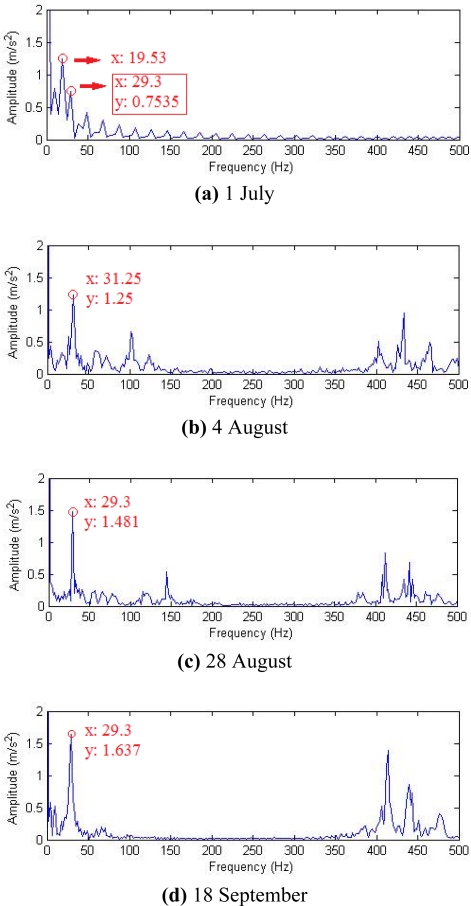
Demodulation spectra with composite dictionary multi-atoms matching.

Historical data on 1 July, 4 August, 28 August and 18 September were reviewed for analysis, and their waveforms and frequency spectra are shown in Figure 18. No impact features can be seen in the waveforms, which contain a lot of noise due to the industrial operating conditions. After the harmonic component was separately extracted through composite dictionary multi-atoms matching analysis, the resulting demodulation spectra are shown in Figure 19. From the spectrogram, the fault characteristic frequencies at 29.3 Hz or 31.25 Hz (with a certain deviation from the actual fault characteristic frequency 30.1 Hz due to the frequency resolution) could be clearly seen. In fact, the rotation frequency of shaft II was fluctuant in a small area at the project site, not exactly 30.1 Hz, and 30.1 Hz was a theoretical reference value. Besides, the amplitude became increasingly large at 0.7353, 1.225, 1.481 and 1.637. On 1 July, however, the fault characteristic frequency at 29.3 Hz was not so clear, and the amplitude was general, indicating that the gear faults on Shaft II of the bevel box had become prominent and developed since 4 August or even earlier. The dismantled finishing support 22 was checked in November and noticed that gear Z5 (with 31 teeth) on shaft II of the bevel box was broken, as shown in Figure 17.

**Figure 20.**
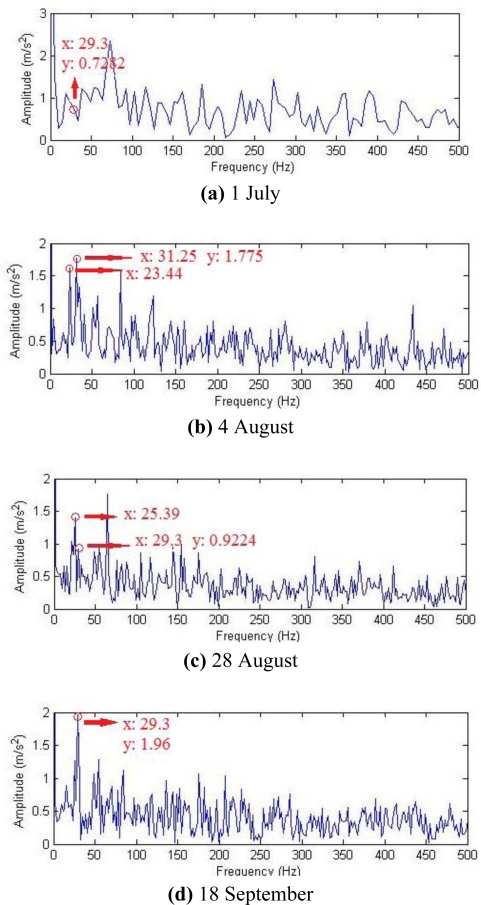
Demodulation spectra with single-atom matching.

If the same four groups of signals after hard threshold were reconstructed with the impulse time-frequency dictionary single-atom matching, then demodulation spectra could be obtained through demodulation, as shown in Figure 20. It was clear that all the fault characteristic frequencies in the three groups of data other than those that could be clearly seen on 18 September suffered interference from the intense noise, and the fault diagnosis judgment cannot be exactly decided. While fault characteristic frequencies could be detected with the composite dictionary multi-atom matching on 4 August or even earlier, which provided earlier prediction and prevention information for the occurrence and development of gear faults.

## 8. Conclusions

MP is a classic algorithm for sparse decomposition. However, it has certain drawbacks in the sparse expression of complicated and non-stationary signals due to rather singleness in the matching between the atoms in an atom dictionary and signals as well as the enormous amount of computation. Aiming at resolving this problem, a new sparse decomposition and reconstruction algorithm is proposed based on the composite dictionary multi-atom matching pursuit in this paper. The algorithm constituted a composite dictionary combining the impulse time-frequency dictionary and the Fourier dictionary to extract the impulse component on the basis of the structural characteristics of gear fault signals.

With the principle of threshold de-noising introduced in the reconstruction algorithm with composite dictionary multi-atom matching, the threshold was set. A hard threshold was performed on matching coefficients of all orders acquired with the decomposition algorithm, *i.e.*, the matching coefficients being less than a threshold were set to be zero for reconstruction. According to the analysis results of data that were proved by the adoption of kurtosis indices and impulse indices, the effect of impulse signal extraction after hard threshold was better. Under intensified noise, the signal impulse effects after multi-atoms matching and related hard threshold were significantly improved compared with primary noising signals while those with single-atom threshold and hard threshold were not enhanced much. In other words, composite dictionary multi-atoms matching achieve a better analytical effect than single-atom matching under the influence of intense noise.

The data analysis showed that increasing data length would cause the computing amount of the decomposition algorithm and the running time of the program to be significantly increased. Accordingly, the algorithm was improved, *i.e.*, a data sequence was evenly segmented by 512 points. The decomposition, threshold processing and reconstruction with multi-atom matching were conducted on each section, respectively. The data analysis proved that the calculation efficiency was significantly enhanced with the improved algorithm compared with the primary one. Further research is currently undergoing to analyze the more complex gearbox signals and improve the proposed method and make it more feasible.

## Figures and Tables

**Table 1.** Comparison results of kurtosis indices and impulse indices.

**Signal**	**Noising signal**	**Multi-atoms non-threshold**	**Multi-atoms hard threshold**	**Single-atom non-threshold**	**Single-atom hard threshold**

Kurtosis index	3.61	5.11	14.81	4.30	11.36
Impulse index	4.77	6.21	14.07	5.80	12.35

**Table 2.** Influences of noise intensity on the effects of impulse signal extraction with multi-atoms matching or single-atom matching.

	***SNR* = −0.72 dB**		***SNR*= −6.60 dB**		***SNR* = −10.21 dB**	
	Kurtosis index	Impulse index	Kurtosis index	Impulse index	Kurtosis index	Impulse index
**Noising signal**	3.61	4.77	2.76	3.22	3.18	4.10
**Multi-atoms non-threshold**	5.11	6.21	4.39	5.15	3.79	4.92
**Multi-atoms hard threshold**	14.81	14.07	9.99	10.82	8.30	8.92
**Single-atom non-threshold**	4.30	5.80	2.83	3.50	3.97	6.24
**Single-atom hard threshold**	11.36	12.35	6.24	6.94	4.22	5.81

**Table 3.** Comparison of iteration times and running time of initial algorithm and improved algorithm.

**Number of data points**	**Frequency resolution (Hz)**	**Initial decomposition algorithm**		**Segmented decomposition algorithm**	

		**Iteration times**	**Running time (s)**	**Iteration times**	**Running time (s)**

512	30	50	128	54	141
1,024	15	106	470	117	294
2,048	7.5	234	2,210	235	587

**Table 4.** The sampling parameters of historical data in each group.

	**1 July**	**4 August**	**28 August**	**18 September**

**Sampling length**	2,048	2,048	2,048	2,048
**Sampling frequency**	10 kHz	4 kHz	4 kHz	4 kHz
